# CircSMARCA5 Inhibits Migration of Glioblastoma Multiforme Cells by Regulating a Molecular Axis Involving Splicing Factors SRSF1/SRSF3/PTB

**DOI:** 10.3390/ijms19020480

**Published:** 2018-02-06

**Authors:** Davide Barbagallo, Angela Caponnetto, Matilde Cirnigliaro, Duilia Brex, Cristina Barbagallo, Floriana D’Angeli, Antonio Morrone, Rosario Caltabiano, Giuseppe Maria Barbagallo, Marco Ragusa, Cinzia Di Pietro, Thomas Birkballe Hansen, Michele Purrello

**Affiliations:** 1 Department of Biomedical and Biotechnological Sciences—Section of Biology and Genetics, University of Catania, 95123 Catania, Italy; dbarbaga@unict.it (D.B.); caponnettoangela@gmail.com (A.C.); matildecirnigliaro@gmail.com (M.C.); duiliabrex@gmail.com (D.B.); barbagallocristina@gmail.com (C.B.); floriana.dangeli@hotmail.it (F.D.); mragusa@unict.it (M.R.); dipietro@unict.it (C.D.P.); 2 Department of Medical, Surgical Sciences and Advanced Technologies and Biotechnological Sciences G.F. Ingrassia, University of Catania, 95123 Catania, Italy; morant592@libero.it (A.M.); rosario.caltabiano@unict.it (R.C.); gbarbagallo@unict.it (G.M.B.); 3 Multidisciplinary Research Center on Brain Tumors Diagnosis and Therapy, University of Catania, 95123 Catania, Italy; 4 Department of Molecular Biology and Genetics (MBG), Aarhus University, 8000 Aarhus C, Denmark; 5 Interdisciplinary Nanoscience Center (iNANO), Aarhus University, 8000 Aarhus C, Denmark

**Keywords:** circRNAs, glioblastoma multiforme, cell migration, splicing, RNA binding proteins

## Abstract

Circular RNAs (circRNAs) have recently emerged as a new class of RNAs, highly enriched in the brain and very stable within cells, exosomes and body fluids. To analyze their involvement in glioblastoma multiforme (GBM) pathogenesis, we assayed the expression of twelve circRNAs, physiologically enriched in several regions of the brain, through real-time PCR in a cohort of fifty-six GBM patient biopsies and seven normal brain parenchymas. We focused on hsa_circ_0001445 (circSMARCA5): it was significantly downregulated in GBM biopsies as compared to normal brain tissues (*p*-value < 0.00001, student’s *t*-test), contrary to its linear isoform counterpart that did not show any differential expression (*p*-value = 0.694, student’s *t*-test). Analysis of a public dataset revealed a negative correlation between the expression of circSMARCA5 and glioma’s histological grade, suggesting its potential negative role in the progression to malignancy. Overexpressing circSMARCA5 in U87MG cells significantly decreased their migration, but not their proliferation rate. In silico scanning of circSMARCA5 sequence revealed an enrichment in binding motifs for several RNA binding proteins (RBPs), specifically involved in splicing. Among them, serine and arginine rich splicing factor 1 (SRSF1), a splicing factor known to be a positive controller of cell migration and known to be overexpressed in GBM, was predicted to bind circSMARCA5 by three different prediction tools. Direct interaction between circSMARCA5 and SRSF1 is supported by enhanced UV crosslinking and immunoprecipitation (eCLIP) data for SRSF1 in K562 cells from Encyclopedia of DNA Elements (ENCODE). Consistently, U87MG overexpressing circSMARCA5 showed an increased expression of serine and arginine rich splicing factor 3 (SRSF3) RNA isoform containing exon 4, normally skipped in a SRSF1-dependent manner, resulting in a non-productive non-sense mediated decay (NMD) substrate. Interestingly, SRSF3 is known to interplay with two other splicing factors, polypyrimidine tract binding protein 1 (PTBP1) and polypyrimidine tract binding protein 2 (PTBP2), that positively regulate glioma cells migration. Collectively, our data show circSMARCA5 as a promising druggable tumor suppressor in GBM and suggest that it may exert its function by tethering the RBP SRSF1.

## 1. Introduction

Circular RNAs (circRNAs) have recently been established as a comprehensive class of RNAs generated by non-linear back-splicing [1,2,3,4,5,6]. CircRNAs are characterized by covalently joined 5′- and 3′-ends: this peculiarity renders them intrinsically resistant to degradation mediated by exonucleases and, consequently, more stable than linear isoform counterparts, both inside and outside the cells [7]. CircRNAs show species-, cell type- and developmental stage-specific expression patterns [8,9,10]: in animals, they are highly enriched in neural tissues [11,12] and furthermore detectable in several biological fluids, thereby useful as non-invasive robust biomarkers [13,14,15,16,17]. Notwithstanding dysregulation of circRNA expression has been highlighted in several types of cancer, most deregulated circRNAs remain functionally undisclosed till today [18,19,20]. Most studies describe circRNAs as microRNA (miRNA) sponges, however, for some of them, a role as modulators of RNA binding proteins (RBPs) has also been suggested [21,22,23,24,25,26,27,28]. To pinpoint functional involvement of circRNAs in glioblastoma multiforme (GBM), we chose twelve circRNAs abundantly expressed in human brain and whose expression is known to be modulated during biological processes linked to oncogenesis (e.g., differentiation, epithelial-to-mesenchymal transition, etc.). In this study, we focused on circSMARCA5, the most downregulated circRNA (among the twelve analyzed) in GBM samples as compared to healthy brain parenchyma, and characterized it as a novel tumor-suppressor, regulating the migration of GBM cells. Our data indicate that circSMARCA5 may exert its function by modulating the RNA binding protein serine and arginine rich splicing factor 1 (SRSF1), a known oncoprotein involved in positive regulation of cell migration [29].

## 2. Results

### 2.1. CircRNA Expression Profile

**Figure 1:**
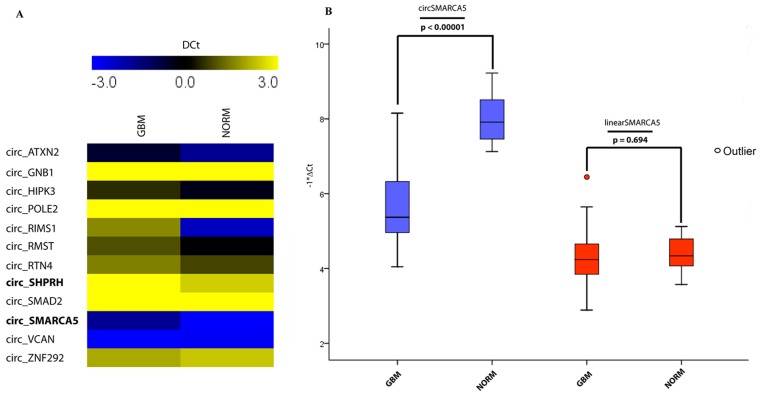
Expression of candidate circRNAs in GBM and control (NORM) samples. (**A**) Heat map representing the expression profile of twelve candidate circRNAs in GBM and control samples. Expression values (reported as DCt) higher and lower than those of the control are shown in yellow and blue, respectively. circSHPRH and circSMARCA5 are in bold, due to their significant downregulation (*p*-value circSHPRH = 0.015, *p*-value circSMARCA5 = 0.017, *n* = 5, Student’s *t*-test). (**B**) Box-plots representing the expression of circSMARCA5 and linearSMARCA5 in GBM and control samples. Expression values are reported as −DCt relative to (mRNA) *TBP* (*p*-values are shown in the figure, *n*_GBM_ = 56, *n*_NORM_ = 7, Student’s *t*-test).

Expression profile of twelve selected circRNAs in a training set of five fresh-frozen GBM samples matched with adjacent non-tumor tissues revealed a significant downregulation of hsa_circ_0001649/circSHPRH (median fold-change = −2.18, *p*-value = 0.015) and hsa_circ_0001445/circSMARCA5 (median fold-change = −2.42, *p*-value = 0.017) (Figure 1A). Here, we focus on circSMARCA5, because of its higher abundance in several regions of the brain as well in GBM biopsies with respect to circSHPRH, as reported by circBase and by Song et al. [30]. Downregulation of circSMARCA5 was confirmed in a test set cohort of fifty-six formalin-fixed paraffin embedded (FFPE) GBM biopsies and seven controls (median fold-change = −5.83, *p*-value < 0.00001). Interestingly, the expression of the linear isoform counterpart did not vary, suggesting a specific dysregulation of the circular isoform (Figure 1B). CircSMARCA5 down expression was also observed in five GBM cell lines with respect to healthy astrocytes, suggesting a contribution of this cell type to circSMARCA5 dysregulation in GBM (Figure S1) (see Section 3).

2.2. circSMARCA5 Expression Is Linked to Progression through Malignancy

**Figure 2:**
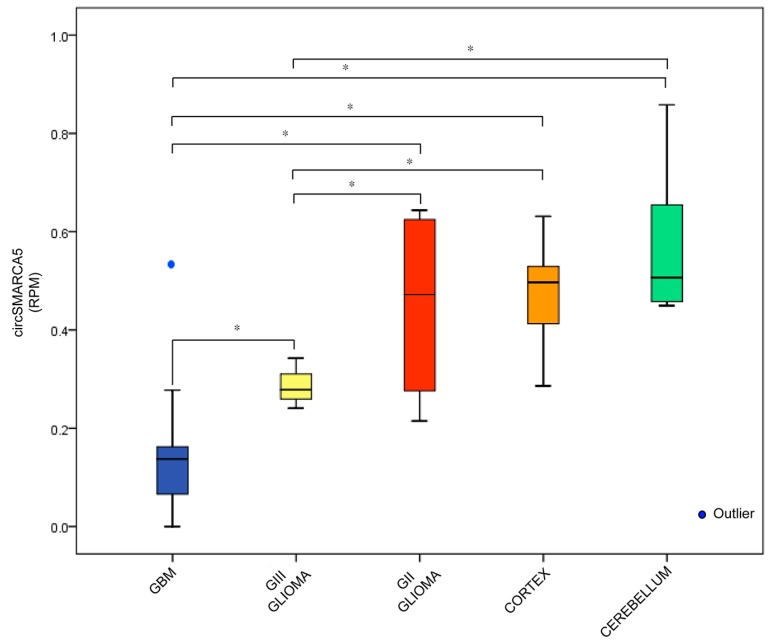
Correlation between circSMARCA5 expression and glioma grade malignancy. Box-plot representing circSMARCA5 expression in three grade tumor groups (GBM, Grade III—GIII- and Grade II—GII-gliomas) and two different control groups (Cortex and Cerebellum). Expression values are reported as reads per million mapped reads (RPM) (* *p*-value < 0.05, ANOVA test).

Data reported in the previous paragraph were reproducible in an independent dataset made of twenty GBM, three grade III glioma, four grade II glioma, thirteen normal brain cortex and six normal cerebellum [30]: specifically, this dataset showed that a decreased expression of circSMARCA5 matched with an increase in glioma grade malignancy (Figure 2).

### 2.3. CircSMARCA5 Cloning and Expression in GBM Cells

pcDNA3_circSMARCA5 transfected in U87MG cells produced the circular isoform of SMARCA5, as revealed by Northern blotting (Figure S2A). The introns flanking the two exons of *SMARCA5* pre-mRNA involved in circularization contain a 132 nucleotides long conserved inverted repeat (cloned within pcDNA3_circSMARCA5 vector) with a 77% of complementarity that is most probably involved in circSMARCA5 biogenesis (Figure S2B). Based on our analyses, a very small (30 nucleotides long), probably not functional, inverted repeat appeared in genomes of lagomorpha and rodentia for the first time during evolution, whereas a longer, most probably functional, inverted repeat emerged in primate genomes (Table S1). Consistently, circSMARCA5 has not been annotated in mouse according to CircBase, suggesting that circSMARCA5 is primate specific and that biogenesis depends on the long inverted repeat.

### 2.4. CircSMARCA5 Inhibits GBM Cells Migration without Altering Cell Viability

**Figure 3:**
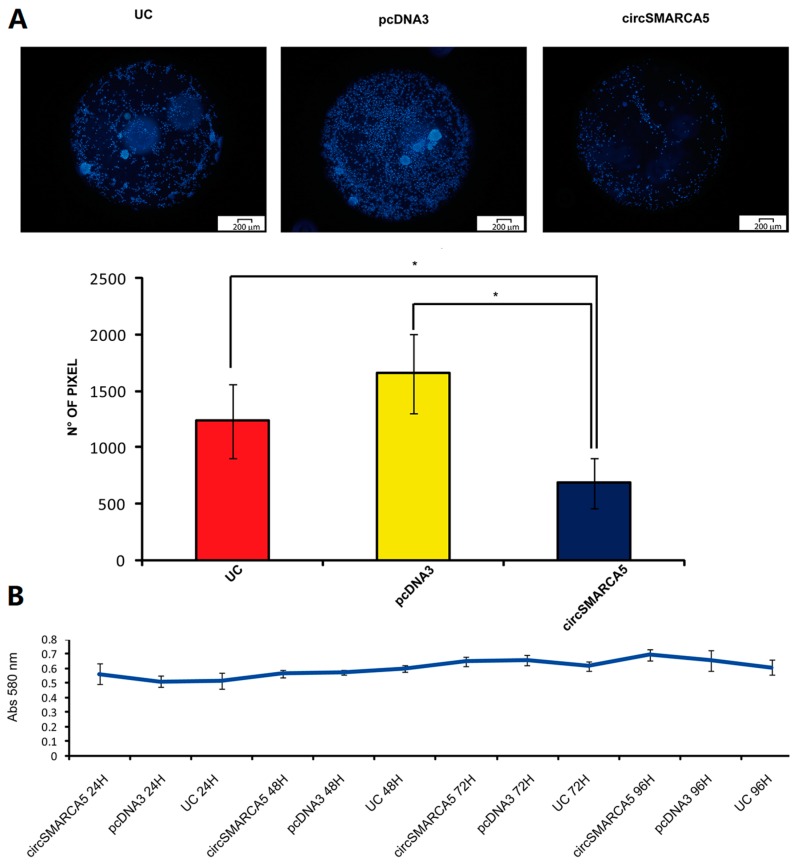
Effects of circSMARCA5 overexpression on migration and viability of U87MG cells. (**A**) Migration of untransfected (UC), pcDNA3 and pcDNA3_circSMARCA5 (circSMARCA5) transfected U87MG cells. Cells were stained with Hoechst^®^ 33342 at a final concentration of 5 µg/mL (upper panel). Quantitative data are reported as the number of pixel within the detection area (lower panel, * *p*-value < 0.05, *n* = 3, Student’s *t*-test). (**B**) Viability of untransfected (UC), pcDNA3 and pcDNA3_circSMARCA5 (circSMARCA5) transfected U87MG cells. Data are reported as baseline-corrected absorbance at 580 nm (*n* = 6).

Overexpression of pcDNA3_circSMARCA5 in U87MG revealed significant decrease of migration rate as compared to negative control (U87MG transfected with pcDNA3) (average fold change = −2.41, *p*-value = 0.015) (Figure 3A). Viability of U87MG appeared not to be affected under the same experimental conditions (Figure 3B).

### 2.5. CircSMARCA5 Is Predicted to Function as Modulator of Several RBPs

**Figure 4:**
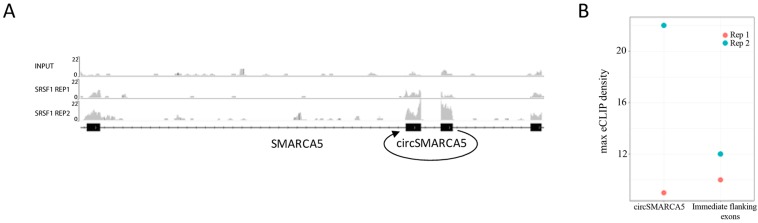
SRFS1 eCLIP data from ENCODE on K562 cells. (**A**) eCLIP read density within the SMARCA5 gene locus (chr4:144461450-144466050, hg19), containing the circSMARCA5 and the immediate flanking exons, visualized by the Integrative Genomics Viewer (IGV). (**B**) Max read density obtained on circSMARCA5 and in the immediate flanking exons (as shown in a) from SRSF1 eCLIP replicates 1 and 2.

To investigate the mechanism through which circSMARCA5 may exert its function, we scanned its sequence for motifs predicted to be bound by miRNAs or RBPs. Probably due to the short length (only 269 nucleotides), no significant enrichment of miRNA binding sites was observed. On the contrary, circSMARCA5 appeared to be enriched in RBP binding sites. Among the RBPs, SRSF1 was predicted to bind circSMARCA5 at multiple sites, by three different tools (Table S2). This prediction was also supported by enhanced UV crosslinking and immunoprecipitation (eCLIP) data on K562, available in Encyclopedia of DNA Elements (ENCODE) (Figure 4).

### 2.6. Splicing of Serine and Arginine Rich Splicing Factor 3 (SRSF3) Is Regulated by CircSMARCA5

**Figure 5:**
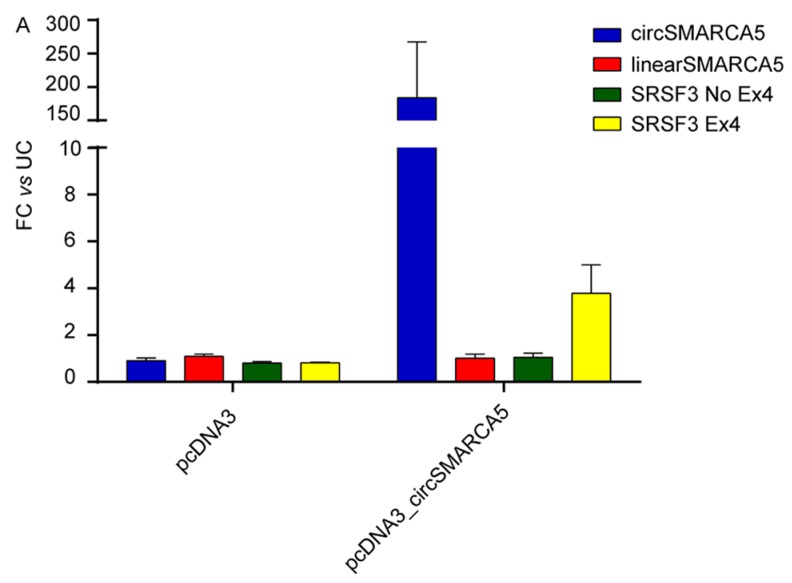

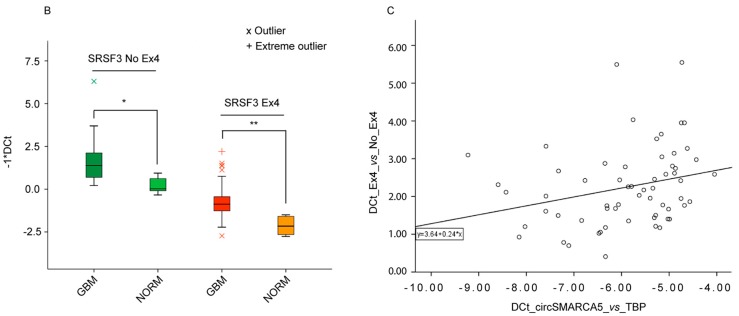
circSMARCA5 stimulates inclusion of exon 4 within *SRSF3* mRNA. Graphs representing the expression of the mRNA of the splicing factor *SRFS3* in: (**A**) U87MG overexpressing circSMARCA5; and (**B**,**C**) GBM biopsies. (**A**) U87MG overexpressing circSMARCA5 show a significant increased expression of *SRSF3* containing exon 4 (*SRSF3 Ex4*) (** *p*-value < 0.01, *n* = 3, Student’s *t*-test). Data are reported as fold-change (FC) versus untransfected cells (UC). (**B**) Both *SRSF3 No Ex4* and *SRSF3 Ex4* are significantly overexpressed in GBM biopsies (GBM) with respect to normal brain parenchyma (NORM) (* *p*-value < 0.05; ** *p*-value < 0.01, *n*_GBM_ = 56, *n*_NORM_ = 7, Student’s *t*-test). Expression values are reported as −DCt relative to (mRNA) *TBP.*(**C**) Correlation between *SRSF3 Ex4*/*SRSF3 No Ex4* ratio and circSMARCA5 (*r*-value = 0.36, *p*-value = 0.004, Spearman’s correlation test). Data are represented as DCt relative to (mRNA) *TBP* in a scatter plot.

To investigate the possible functional role of circSMARCA5 as regulator of splicing, we transfected U87MG cells with pcDNA3_circSMARCA5 vector and analyzed the expression of two isoforms of (mRNA) *SRSF3*, with or without exon 4 skipped. It is known that the inclusion of exon 4 within *SRSF3* pre-mRNA is controlled by SRSF1 during splicing [31]. We observed an increase in the expression specifically of the (mRNA) *SRSF3* isoform including exon 4 in U87MG overexpressing circSMARCA5 (Figure 5A). These data are consistent with a significant upregulation of (mRNA) *SRSF3* with skipped exon 4 (*SRSF3 No Ex4*) in the same cohort of GBM biopsies exhibiting a significant downregulation of circSMARCA5 (Figure 5B). Unexpectedly, (mRNA) *SRSF3* including exon 4 (*SRSF3 Ex4*) was also upregulated in GBM biopsies (Figure 5B) but, consistently with data obtained in U87MG cells overexpressing circSMARCA5, the *SRSF3 Ex4/SRSF3 No Ex4* ratio positively correlated with the expression of circSMARCA5 in GBM biopsies (Figure 5C) (see Section 3).

## 3. Discussion

CircRNAs are on the forefront of basic and applied research but, notwithstanding the accumulating evidence of their potential interest as critical regulators of gene expression, only a few studies describe the molecular mechanisms through which they work. In this paper, we focused on hsa_circ_0001445/circSMARCA5, an exonic 269-nucleotide-long circRNA, highly enriched in human brain (http://www.circbase.org/). We found that its expression decreases in GBM cells with respect to normal brain parenchyma, independently from the expression level of the linear isoform. Moreover, astrocytes (which is the most representative cell type in GBM biopsies) most likely contribute to altered expression of circSMARCA5 in GBM (Figure S1), although it is not possible to exclude that also other cell types—e.g., Tumor Associated Macrophages (TAMs)—may impact on circSMARCA5 dysregulation. This result matches with a previously described increase of circular to linear transcript ratios, observed during differentiation of cardiomyocytes [32] and, together with the inverse relationship between circSMARCA5 expression and glioma malignancy grade, this collectively suggests a functional involvement in GBM. These data, together with Receiver Operating Characteristic (ROC) curves data (Figure S3), also suggest circSMARCA5 as a candidate GBM biomarker, although the use of the term biomarker is referred to a scanty cohort of patients at present and should be re-evaluated in a wider case-control study. To characterize the effects of circSMARCA5 expression, we first cloned its sequence, together with a segment of the introns upstream and downstream the exons that circularize, into an expression vector. Flanking intronic sequences contained an inverted repeat of 132 nucleotides showing 77% complementarity: these sequences are most probably responsible for the biogenesis of circSMARCA5 and are conserved among primates (Figure S4), suggesting a primate-specific expression of this circRNA. According to functional analysis, circSMARCA5 negatively regulates GBM cell migration. Based on further in vitro and in silico characterization, we also speculate that circSMARCA5 may exert its function by disrupting splicing of GBM cells, specifically by modulating the SRSF1 splicing factor. This hypothesis is supported by eCLIP data from ENCODE (Figure 4) as well as by the circSMARCA5-mediated changes in (mRNA) *SRSF3* splicing pattern (Figure 5A) and by data on GBM biopsies (Figure 5B,C). Based on our data of circSMARCA5 modulation (Figure 5A) and according to Jumaa et al. and Jihua et al. [31,33], we hypothesize that circSMARCA5 downregulation in GBM biopsies, associated with upregulation of SRSF1 (Figure S5), may promote the skipping of exon 4 in *SRSF3* pre-mRNA. At the same time, increased levels of functional SRSF3 protein may determine the increased expression of (mRNA) *SRSF3* containing exon 4 (which results in a non-productive non-sense mediated decay (NMD) substrate [34,35]), in a self-regulatory manner (Figure 5B). Despite the increased expression of both (mRNA) *SRSF3* isoforms in GBM biopsies compared to normal brain parenchyma, we observe a significant positive correlation between *SRSF3 Ex4/SRSF3 No Ex4* ratio and circSMARCA5 expression (Figure 5C). These data support our hypotheses that circSMARCA5 may indirectly regulate the expression of (mRNA) *SRSF3* isoforms, by tethering SRSF1, and, in turn, functional SRSF3 protein (synthesized from *SRSF3 No Ex4*) may positively regulate the expression of *SRSF3 Ex4*, which is normally expressed at very low levels within cells (Figure S6). Often the networks involved in gene regulation within cells are intricate, so we do not exclude that other nodes (in addition to circSMARCA5) may contribute in regulating the expression of (mRNA) *SRSF3* isoforms within GBM cells. Interestingly, SRSF1 is known to regulate a plethora of biological functions, further to splicing, within cells [36] and is located in the middle of a self-regulatory network involving several splicing factors such as PTBP1 and SRSF3 that act as known oncoproteins in different types of cancer [37]. Most specifically, splicing pattern of (mRNA) *SRSF3* is defined by SRSF1 and SRSF3 itself regulates the expression of PTBP1 whose overexpression is known to positively regulate GBM cells migration [38]. Conclusively, circSMARCA5 is significantly downregulated in GBM and it may exert its function by modulating the activity of SRSF1 with subsequent effects on SRSF3 and PTBP1 splicing and expression. An in-depth study of the interaction between circSMARCA5 and SRSF1 and its downstream network within GBM cells and to strengthen the possible use of circSMARCA5 as GBM biomarker by extending the case-control study, will represent the natural continuation of this project.

## 4. Materials and Methods

### 4.1. GBM Specimens and Cell Lines

GBM biopsies from five fresh-frozen (training set) and fifty-six FFPE (test set) samples were diagnosed by at least three experienced pathologists, according to the 2007 WHO criteria [39]. Each sample contained more than 90% of cancer tissue. Normal brain parenchyma was obtained from area surrounding the tumor that appeared negative to 5-aminolevulinic acid (5-ALA) fluorescence during surgery, from seven patients. Written informed consent was received from participants prior to inclusion in the study. Absence of infiltration of cancer cells within normal brain parenchyma was also confirmed by microscopy analysis performed by at least three experienced pathologists. Additionally, the commercially available FirstChoice^®^ Human Brain Reference RNA (Ambion, Austin, TX, USA) and Human Astrocyte Total RNA (ScienCell Research Laboratories^®^, San Diego, CA, USA) were used as control healthy tissues. Age, sex and clinical features of the analyzed tumor samples and healthy controls are reported in Table 1. Human GBM cell lines A172, CAS1, DBTRG, SNB19 and U87MG were cultured as previously described [19].

### 4.2. Immunohistochemistry

Immunohistochemical analysis, incubation with biotinylated anti-rabbit secondary antibody and immunoreaction visualization were performed as previously described [40]. Rabbit polyclonal antibody against GFAP (DAKO, Glostrup, Denmark) was used at a dilution 1:7000. GFAP-positive (GFAP^+^) cells percentage was evaluated in the highest immunoreactivity fields. It was determined by dividing the number of positive staining cells by 1000 cells. Cells were considered to be positive if there was any cytoplasmic staining present.

### 4.3. Constructs and Transfections

A 1450 bp PCR amplified fragment of the human genome, comprising exons 15 and 16 of SMARCA5 (the two exons that circularize generating circSMARCA5), the intron between them and 440 bp upstream exon 15 and 546 bp downstream exon 16, was inserted in pcDNA3 vector (Figure S7) after digestion with BamHI and ApaI. U87MG cells were transfected with Lipofectamine 2000 (Thermo Fisher Scientific, Waltham, MA, USA), according to supplier’s protocol. Transfection efficiencies of more than 80% were achieved.

### 4.4. RNA Extraction

Within 30 min from surgical resection, biopsies were collected in sterile tubes on ice, washed in cold sterile PBS to eliminate any blood residue and stored in RNAlater^®^ Stabilization Solution (Thermo Fisher Scientific) for 16 h at 4 °C. Biopsies were then either fresh-frozen or formalin-fixed and paraffin embedded and stored until their processing. RNA from fresh-frozen and FFPE biopsies was extracted by using TRIzol^®^ and RecoverAll^®^ kits (Thermo Fisher Scientific), respectively, according to manufacturer’s instructions and quantified both through spectrophotometer and Qubit™ fluorometer (Thermo Fisher Scientific).

### 4.5. CircRNA Candidates Selection and Primer Design

Manual curation of scientific papers, allowed us to select candidate circRNAs, based on their association with at least one of the following biological processes: (i) regulation of neuronal differentiation; (ii) epithelial-to-mesenchymal (EMT) transition; and (iii) cell proliferation (specifically referred to GBM cells or as general process). All selected circRNA transcripts had to be expressed (and preferentially enriched) in human brain, according to RNA Seq data stored in circBase (http://www.circbase.org/). Candidate circRNAs assayed in this study are reported in Table 2. Both convergent (detecting linear isoforms) and divergent (detecting circular RNAs) primers were designed through the tool NCBI primer blast. Primer pairs were tested in silico and all those primer pairs that recognized more than one circular isoforms produced by the same host gene were discarded. Primer pairs used in this study are reported in Table S3.

### 4.6. Northern Blotting

Ten micrograms of whole cell RNA were loaded on a 1.2% denaturing agarose gel. Transfer to the membrane, labelling of probes, hybridization and detection were conducted as previously described [21].

### 4.7. qRT-PCR

Total RNA was reverse-transcribed in cDNA and amplified using gene-specific primers either in two steps, by MLV-RT (Thermo Fisher Scientific) using random hexamer primers and LightCycler^®^ 480 SYBR Green I Master (Roche Molecular Systems, Inc., Pleasanton, CA, USA), or in one step, using RNA-to-Ct™ 1-Step Kit (Thermo Fisher Scientific). Quantitative PCR was performed on either 7900HT Fast Real-Time PCR System (Thermo Fisher Scientific) or LightCycler 480 (Roche Molecular Systems, Inc.), according to supplier’s protocol. *TBP* and *GAPDH* were used as endogenous control genes. The relative amount of gene expression and the fold change for each transcript were calculated according to 2^−ΔΔ*C*t^ method [41].

### 4.8. Cell Migration Assay

Cell migration was assessed using Oris™ Cell Migration Assay (Platypus Technologies, Madison, WI, USA), as previously described [42]. Briefly, thirty-five thousand cells per well were seeded in the migration ninety-six wells plate with the detection mask attached at the bottom and the stoppers placed. Twenty-four hours after seeding, cells were transfected and grown for other 24 h in a serum free medium. After this time, the stoppers were removed and migration rate was revealed in the detection zone after 24 h by staining the cells with Hoechst^®^ 33342 at a final concentration of 5 µg/mL. Migrated cells were quantified through ImageJ software v. 1.51.

### 4.9. Cell Viability Assay

Cell viability was measured using MTT (3(4,5-dimethylthiazol-2-yl)2,5-diphenyl-tetrazoliumbromide), as previously described [43]. Briefly, twenty-four thousand cells per well were seeded in a ninety-six wells plate, grown for 24 h and then transfected for other 24 h. Viability was assessed reading absorbance at 580 nm 24 h, 48 h, 72 h and 96 h after transfection.

### 4.10. In Silico Analysis

RBP binding site predictions within circSMARCA5 sequence were done by providing the circSMARCA5 FASTA sequence as input in RBPmap [44], ATTRACT (https://attract.cnic.es/index) and catRapid (http://service.tartaglialab.com/page/catrapid_group) online tools, respectively. Inverted repeats were retrieved using EMBOSS einverted tool (http://emboss.bioinformatics.nl/cgi-bin/emboss/einverted). UCSC (https://genome.ucsc.edu/) and ClustalW2 (https://www.ebi.ac.uk/Tools/msa/clustalw2/) were used to assess conservation among sequences.

### 4.11. eCLIP Analysis

Bam-files (accessions ENCFF913MGS, ENCFF374XWQ, and ENCFF833PXB) were downloaded from http://www.encodeproject.org and indexed with samtools. Read densities were visualized using the IGV browser and analyzed using the pysam module in python.

### 4.12 TCGA Data

TCGA GBM RNA-seq data were retrieved from Cancer RNA-Seq Nexus database (http://syslab4.nchu.edu.tw/).

### 4.13 Nomenclature of Genes and Proteins

Genes, mRNAs and proteins are named according to the guidelines set by Human Gene Nomenclature [45].

### 4.14. Statistical Analyses

Unless otherwise specified, statistical significance was assessed by two-tailed student’s *t*-test.

## Figures and Tables

**Table 1:** Clinical data of GBM and control samples.

Type of Samples	N° of Samples	Mean Age (Years ± StdDev)	Sex		Mean Overall Survival (Months)	Preoperative Tumor Volume (mm^3^)
			M	F		
Training set (Fresh-frozen biopsies)	10	60.5 ± 12.1	5	5	19.1 ± 7.2	39.9 ± 11.8
Test set (FFPE biopsies)	56	62 ± 12.7	33	23	17 ± 14.2	28.2 ± 9.2
Normal Brain Parenchyma	7	60.4 ± 11.1	2	5		
FirstChoice^®^ Human Brain Reference Total RNA	1 (commercially available)	68.3 ± 15	13	10		

**Table 2:** Candidate circRNAs.

#	Candidate circRNA (circBase ID)	Parental Gene Symbol	Known Modulation or Function of circRNA Expression in Specific Cell Context	Source (PMID)
1	hsa_circ_0028270	ATXN2	Upregulated during EMT. This circRNA is also highly expressed in several SNC districts (see PMID: 25921068)	25768908
2	hsa_circ_0008702	GNB1	Downregulated during EMT. This circRNA is also highly expressed in several SNC districts (see PMID: 25921068)	25768908
3	hsa_circ_0000284	HIPK3	Involved in cell growth. Highly expressed in normal Brain	27050392
4	hsa_circ_0008002	POLE2	Upregulated during EMT. This circRNA is also highly expressed in several SNC districts (see PMID: 25921068)	25768908
5	hsa_circ_0132250	RIMS1	Downregulated in GBM vs. Normal Brain (another dataset)	26873924
6	hsa_circ_0099634	Rmst	LncRNA Rmst (host gene of the same name circ_Rmst) regulates neuronal differentiation in mouse	25921068
7	hsa_circ_0054598	RTN4	Upregulated during neuronal differentiation both in humans and mice	25921068
8	hsa_circ_0001649	SHPRH	Upregulated during EMT. This circRNA is also highly expressed in several SNC districts (see PMID: 25921068)	25768908
9	hsa_circ_0003694	SMAD2	Upregulated during EMT. This circRNA is also highly expressed in several SNC districts (see PMID: 25921068)	25768908
10	hsa_circ_0001445	SMARCA5	Upregulated during EMT. This circRNA is also highly expressed in several SNC districts (see PMID: 25921068)	25768908
11	hsa_circ_0073237	VCAN	Upregulated in GBM vs. Normal Brain (another dataset)	26873924
12	hsa_circ_0004383	ZNF292	Upregulated in HUVEC under hypoxia. Its silencing reduces endothelial cell proliferation and suppresses tube formation by inhibiting glioma cell proliferation and cell cycle progression in human glioma U87MG and U251 cells	26377962; 27613831

## Supplementary Materials

Supplementary materials can be found at http://www.mdpi.com/1422-0067/19/2/480/s1.
