# Supplementary material for: CircSMARCA5 Inhibits Migration of Glioblastoma Multiforme Cells by Regulating a Molecular Axis Involving Splicing Factors SRSF1/SRSF3/PTB
Source: Int J Mol Sci. 2018 Feb 6;19(2):480. doi: 10.3390/ijms19020480 (PMC5855702; doi:10.3390/ijms19020480)
Supplement: Supplementary file 1 [file ijms-19-00480-s001.docx]

Supplementary files:

**Table S1.** Inverted repeats identified within the introns flanking circSMARCA5 sequences in various species. A window of 300 bp upstream / 300 bp downstream the exons involved in circSMARCA5 formation was given as input in the online tool Einverted (<http://emboss.bioinformatics.nl/cgi-bin/emboss/einverted>) in order to ascertain the presence of inverted repeats.

| **Class** | **Infraclass** | **Order** | **Species** | **Inverted Repeat** |
| --- | --- | --- | --- | --- |
| Mammalia | Eutheria | Primata | *Homo sapiens* (Hsa) | ✓ |
|  |  |  | *Pan troglodytes* (Ptr) | ✓ |
|  |  |  | *Gorilla gorilla* (Ggo) | ✓ |
|  |  |  | *Macaca mulatta* (Mcc) | ✓ |
|  |  | Lagomorpha | *Ochotona princeps* | X |
|  |  |  | *Oryctolagus cuniculus* (Ocu) | ✓ |
|  |  | Rodentia | *Castor canadensis* (Ccan) | ✓ |
|  |  |  | *Mus musculus* (Mmu) | X |
|  |  |  | *Cricetulus griseus* (Cge) | X |
|  |  |  | *Rattus norvegicus* (Rno) | X |
|  | Metatheria | Monotremata | *Ornithorhynchus anatinus* (Oaa) | X |
|  |  | Didelphimorphia | *Monodelphis domestica* (Mdo) | X |
|  |  | Diprotodontia | *Phascolarctos cinereus* | X |
|  |  | Dasyuromorphia | *Sarcophilus harrisii* (Shr) | X |
| Amphibia |  | Anura | *Xenopus leavis* (Xla) | X |
|  |  |  | *Nanorana parkeri* (Npr) | X |
|  |  |  | *Xenopus tropicalis* (Xtr) | X |
| Reptilia |  | Testudines | *Pelodiscus sinensis* (Pss)) | X |
|  |  | Ophidae | *Python bivittatus* (Pbi) | X |
|  |  | Squamata | *Gekko japonicus* (Gja) | X |

**Table S2.** RBPs predicted to bind circSMARCA5 (see Materials and Methods for details).

| **RBP** | **ATTRACT** | **RBPMAP** | ***cat*RAPID** |
| --- | --- | --- | --- |
| SRSF1 | √ | √ | √ |
| TARDBP | √ | √ | Χ |
| CELF1 | √ | Χ | √ |
| CELF2 | √ | Χ | √ |
| CELF4 | √ | Χ | √ |
| CELF5 | √ | Χ | √ |
| CMTR1 | √ | Χ | Χ |
| CPEB4 | √ | Χ | √ |
| DAZAP1 | √ | Χ | Χ |
| DDX58 | √ | Χ | Χ |
| ELAVL1 | √ | Χ | Χ |
| ELAVL2 | √ | Χ | Χ |
| ELAVL4 | √ | Χ | Χ |
| ESRP2 | √ | Χ | √ |
| F2 | √ | Χ | Χ |
| FUS | √ | Χ | √ |
| GRSF1 | √ | Χ | √ |
| HNRNPA0 | √ | Χ | Χ |
| HNRNPA1 | √ | Χ | Χ |
| HNRNPA2B1 | √ | Χ | Χ |
| HNRNPC | √ | Χ | Χ |
| HNRNPD | √ | Χ | Χ |
| HNRNPDL | √ | Χ | Χ |
| HNRNPF | √ | Χ | Χ |
| HNRNPH1 | √ | Χ | Χ |
| HNRNPH2 | √ | Χ | Χ |
| HNRNPH3 | √ | Χ | Χ |
| HNRNPL | √ | Χ | Χ |
| IGF2BP2 | √ | Χ | Χ |
| IGF2BP3 | √ | Χ | Χ |
| KHDRBS1 | √ | Χ | Χ |
| KHSRP | √ | Χ | Χ |
| LIN28A | √ | Χ | Χ |
| MBNL1 | √ | Χ | √ |
| NOVA1 | √ | Χ | √ |
| NOVA2 | √ | Χ | √ |
| NXF1 | √ | Χ | √ |
| OAS1 | √ | Χ | √ |
| PABPC1 | √ | Χ | Χ |
| PABPC3 | √ | Χ | Χ |
| PABPN1 | √ | Χ | Χ |
| PHAX | √ | Χ | √ |
| PIWIL1 | √ | Χ | Χ |
| PPIE | √ | Χ | √ |
| PTBP1 | √ | Χ | √ |
| RBM3 | √ | Χ | √ |
| RBM46 | √ | Χ | √ |
| RBM5 | √ | Χ | Χ |
| RBMS3 | √ | Χ | √ |
| RBMX | √ | Χ | √ |
| RNASEL | √ | Χ | Χ |
| SART3 | √ | Χ | Χ |
| SFPQ | √ | Χ | √ |
| SRP14 | √ | Χ | √ |
| SRP19 | √ | Χ | √ |
| SRP54 | √ | Χ | √ |
| SRP68 | √ | Χ | √ |
| SRSF10 | √ | Χ | Χ |
| SRSF11 | √ | Χ | Χ |
| SRSF2 | √ | Χ | √ |
| SRSF3 | √ | Χ | √ |
| SRSF5 | √ | Χ | √ |
| SRSF6 | √ | Χ | √ |
| SRSF9 | √ | Χ | √ |
| SSB | √ | Χ | Χ |
| TIA1 | √ | Χ | √ |
| TIAL1 | √ | Χ | Χ |
| TRA2A | √ | Χ | √ |
| TRA2B | √ | Χ | √ |
| XPO5 | √ | Χ | Χ |
| YBX1 | √ | Χ | Χ |
| YTHDC1 | √ | Χ | Χ |
| ZFP36 | √ | Χ | Χ |
| ANKHD1 | Χ | √ | Χ |
| BRUNOL4 | Χ | √ | Χ |
| BRUNOL5 | Χ | √ | Χ |
| CNOT4 | Χ | √ | √ |
| CUG-BP | Χ | √ | Χ |
| ESRP2 | Χ | √ | √ |
| FXR1 | Χ | √ | √ |
| G3BP2 | Χ | √ | √ |
| HNRNPC | Χ | √ | Χ |
| HNRNPF | Χ | √ | Χ |
| HNRNPH1 | Χ | √ | Χ |
| HuR | Χ | √ | Χ |
| KHDRBS1 | Χ | √ | Χ |
| KHDRBS2 | Χ | √ | Χ |
| KHDRBS3 | Χ | √ | Χ |
| LIN28A | Χ | √ | Χ |
| MBNL1 | Χ | √ | √ |
| PABPC3 | Χ | √ | Χ |
| PABPC5 | Χ | √ | Χ |
| PABPN1 | Χ | √ | Χ |
| PTBP1 | Χ | √ | √ |
| PUM2 | Χ | √ | Χ |
| RBM24 | Χ | √ | √ |
| RBM3 | Χ | √ | √ |
| RBM41 | Χ | √ | √ |
| RBM46 | Χ | √ | √ |
| RBM5 | Χ | √ | Χ |
| RBMS1 | Χ | √ | √ |
| RBMS3 | Χ | √ | √ |
| SNRNP70 | Χ | √ | Χ |
| SRSF10 | Χ | √ | Χ |
| SRSF2 | Χ | √ | √ |
| SRSF3 | Χ | √ | √ |
| SRSF5 | Χ | √ | √ |
| SRSF7 | Χ | √ | √ |
| TRA2B | Χ | √ | √ |
| TUT1 | Χ | √ | Χ |

**Table S3.** Sequences of primers and probes used in the study.

| **Primer ID** | **Sequence** |
| --- | --- |
| *GAPDH* Fw | GTCAGCCGCATCTTCTTTTG |
| *GAPDH* Rev | GCGCCCAATACGACCAAATC |
| hsa_circ_ATXN2 Fw | TCAGACTTTGTTGTGGTACAGT |
| hsa_circ_ATXN2 Rev | TTGGAGCCCTCTTTTTGCAT |
| hsa_circ_GNB1 Fw | GGGCACAGACTCCAGACAAAT |
| hsa_circ_GNB1 Rev | TGTGAGATCTTAATTCAGAAGGGC |
| hsa_circ_HIPK3 Fw | GGTCGGCCAGTCATGTATCA |
| hsa_circ_HIPK3 Rev | AGGCCATACCTGTAGTACCGA |
| hsa_circ_POLE2 Fw | AGTACTACTAGAGAGCACGTTTTCA |
| hsa_circ_POLE2 Rev | CTGAATTGTACACAAAGCGTGGA |
| hsa_circ_RIMS1 Fw | AGCCTTAGTGCCAAAGTGGT |
| hsa_circ_RIMS1 Rev | TAAGCTTGCTGTTTGACTAAGCTG |
| hsa_circ_RMST Fw | GGGCTAGTTGAGGAATGGCT |
| hsa_circ_RMST Rev | ACTCACTCCATCATCCTGAGA |
| hsa_circ_RTN4 Fw | TGAGTAAAACTTCAGATGAGACCCT |
| hsa_circ_RTN4 Rev | GCAGAGGAGCGTATCACAGG |
| hsa_circ_SHPRH Fw | AAACTGCTGAGAGAAGGGCAG |
| hsa_circ_SHPRH Rev | TCATCAGAGTTCTGACCACAGC |
| hsa_circ_SMAD2 Fw | TGAAGATGGAGAAACAAGTGACC |
| hsa_circ_SMAD2 Rev | AGAGCAAGTGCTGTGTCCATA |
| hsa_circ_SMARCA5 Fw | ACAATGGATACAGAGTCAAGTGTT |
| hsa_circ_SMARCA5 Rev | CCACAAGCCTCCCTTTTGTTTT |
| hsa_circ_VCAN Fw | TGAAACAGATTTCCTGATTGGCA |
| hsa_circ_VCAN Rev | TCATTCGACCTGGTAAATAGATTGC |
| hsa_circ_ZNF292 Fw | GGGTGTGGAAAAACCCGGTA |
| hsa_circ_ZNF292 Rev | TCTGGGGCAAGCCTTTATCC |
| Linear_SMARCA5 Fw | ATGGGTACCAACACTTAGATCTGT |
| Linear_SMARCA5 Rev | AACGTCTCTGACAAAAGCAGC |
| *SMARCA5* Probe | TGCAGTCTTCTTTGCACCTCTTTCCAAAATACCATCGATATCTTCATCAGTGATCTCACT |
| *SRSF3 Ex4* (NR_036610.1) Fw | TCGTCGCCCTCGAGATGAT |
| *SRSF3 Ex4* (NR_036610.1) Rev | GTGGTGAGAAGAGACATGATGGT |
| *SRSF3 No Ex4* (NM_003017.4) Fw | CGTCGCAGATCTCCAAGAAGG |
| *SRSF3 No Ex4* (NM_003017.4) Rev | CCTATCTCTAGAAAGGGACCTGC |
| *TBP* Fw | ACTTGACCTAAAGACCATTGCA |
| *TBP* Rev | GGCTCTCTTATCCTCATGATTACC |


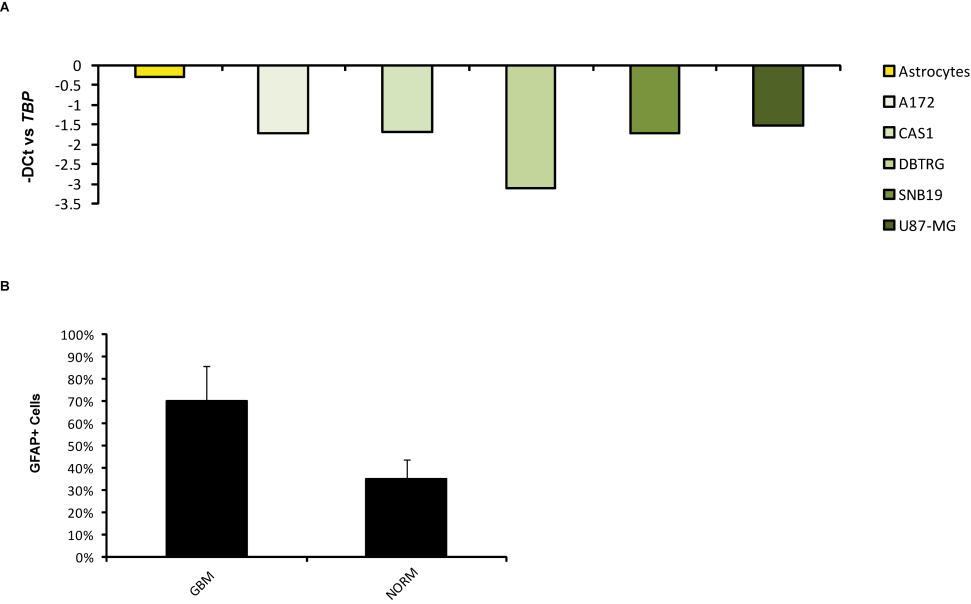


**Figure S1.** (**a**) Expression profile of circSMARCA5 in human astrocytes and GBM cell lines A172, CAS1, DBTRG, SNB19, U87MG. Expression values are reported as –DCt relative to *TBP*; (**b**) percentage of GFAP^+^ cells in GBM (GBM) and normal brain parenchyma (NORM) biopsies. Data are reported as mean ± SD (n_GBM_ = 56, n_NORM_ = 7).


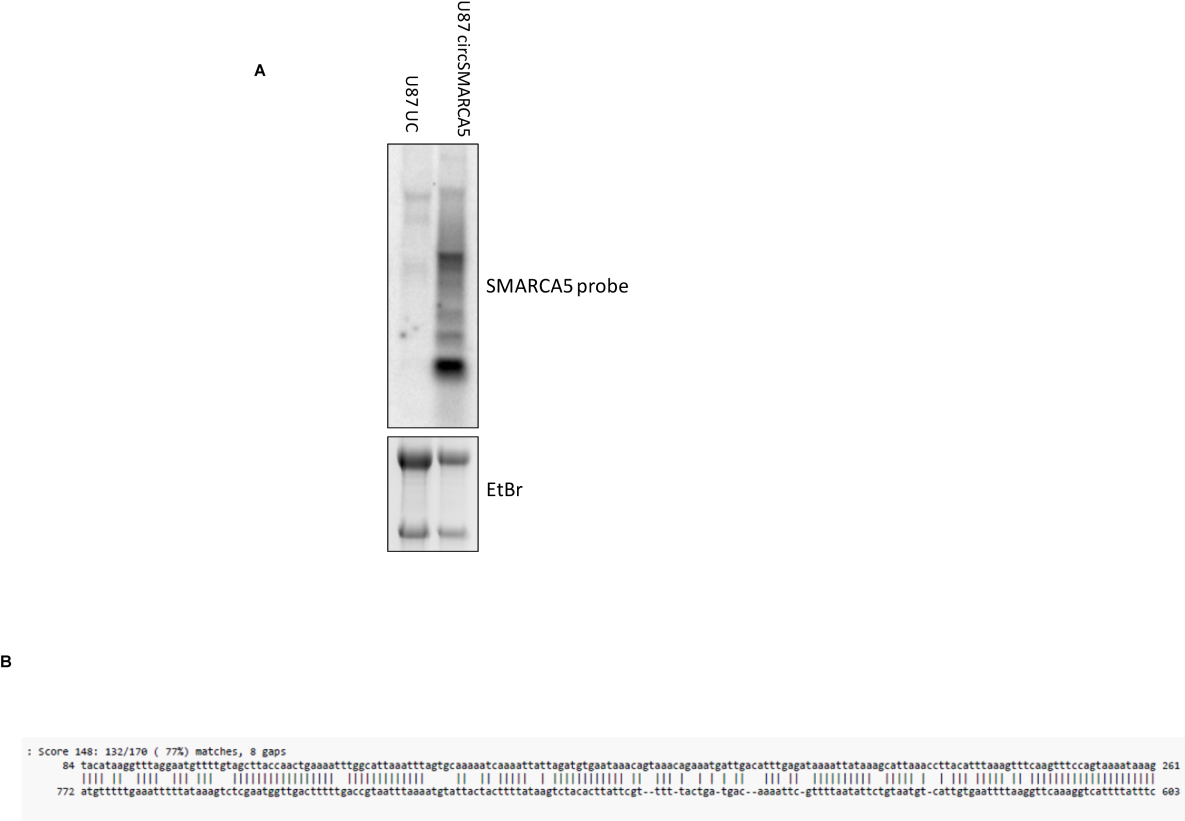


**Figure S2.** (**a**) Northern blot of untransfected (UC) and circSMARCA5-transfected U87MG total RNA with a probe for *SMARCA5*; (**b**) inverted repeat identified by Einverted (<http://emboss.bioinformatics.nl/cgi-bin/emboss/einverted>) within the sequence of introns flanking human circSMARCA5 exons.


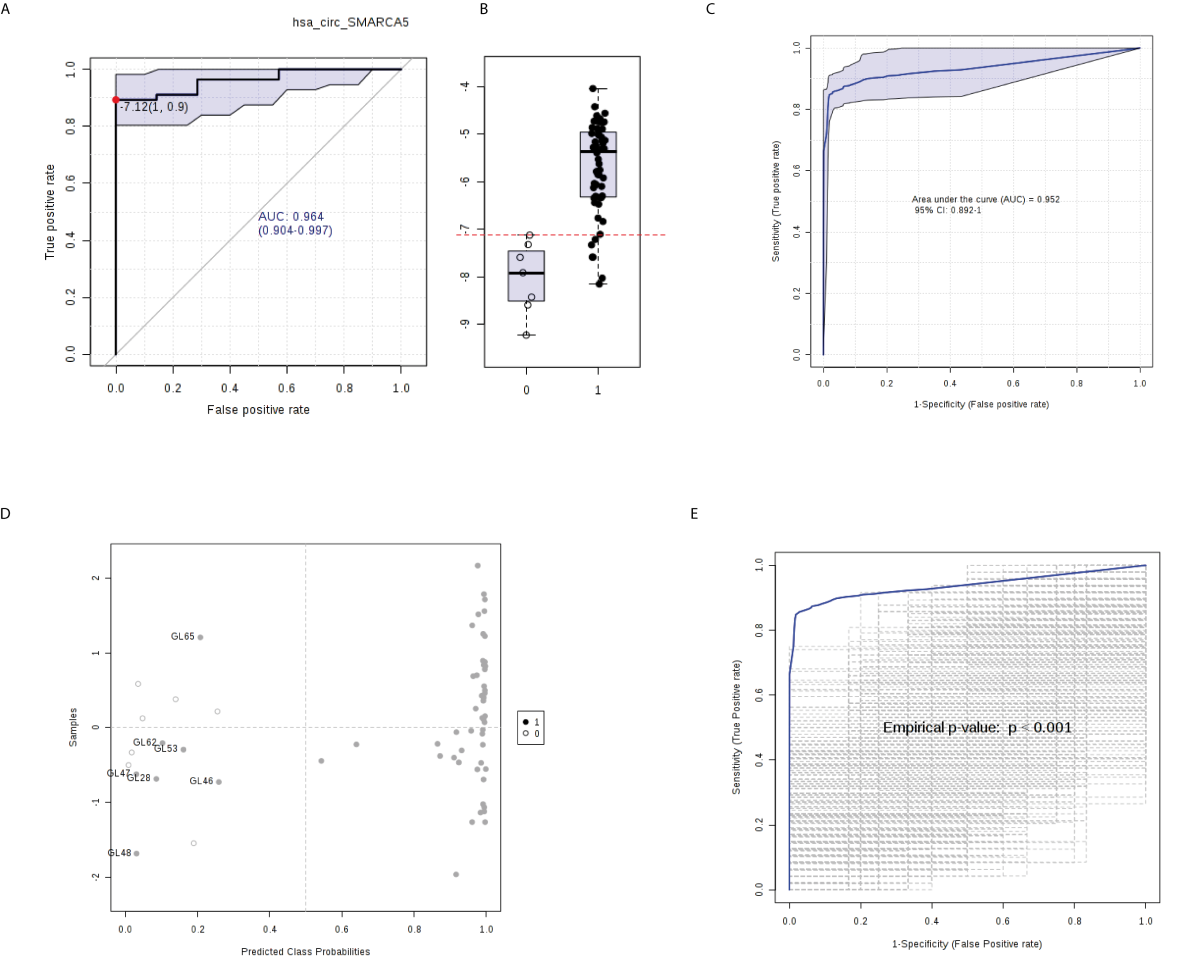


**Figure S3.** ROC curve analysis of circSMARCA5. (**a, b**) The 95% confidence interval is calculated using 500 bootstrappings. Optimal cutoff is showed using farthest to diagonal line (Youden) method; (**c**) Biomarker model. 100 cross validation (CV) were performed and the results were averaged to generate the plot. 95% confidence band is shown. Logistic regression algorithm has been used to generate the curve; (**d**) Average of predicted class probabilities of each sample across the 100 cross-validations. The classification boundary is located at the center (x = 0.5, the dotted line); (**e**) AUROC based on 1000 permutation.

**Figure S4.** Conserved nucleotides within inverted repeat flanking circSMARCA5 sequence. Conservation was assessed by aligning the sequences from twelve primates retrieved in UCSC, through ClustalW. Nucleotides highlighted in yellow and green represent conserved nucleotides within the inverted repeat upstream and downstream circSMARCA5.


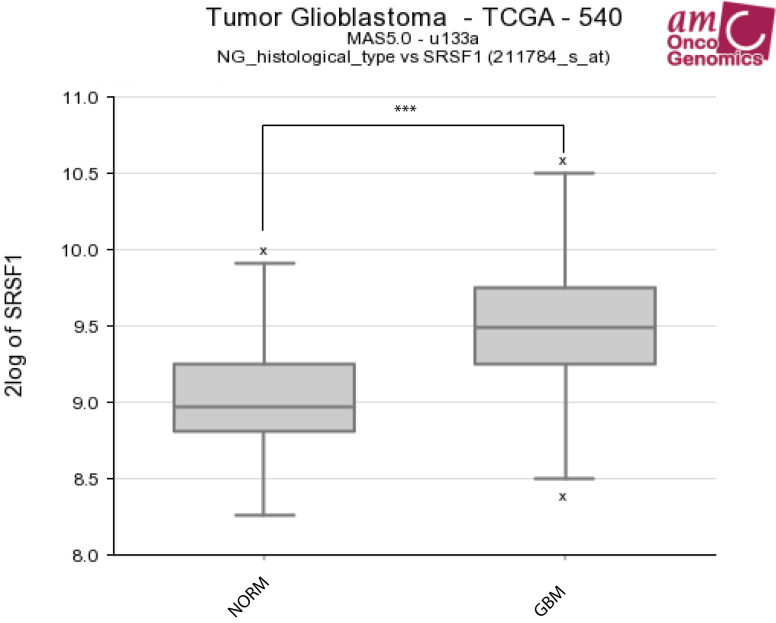


**Figure S5.** Box-plot of the expression of (mRNA) *SRSF1* in GBM and control (NORM) samples (data from Tumor Glioblastoma - TCGA - 540 - MAS5.0 - u133a, analyzed and visualized through R2 platform) (***p-value < 0.001).

**
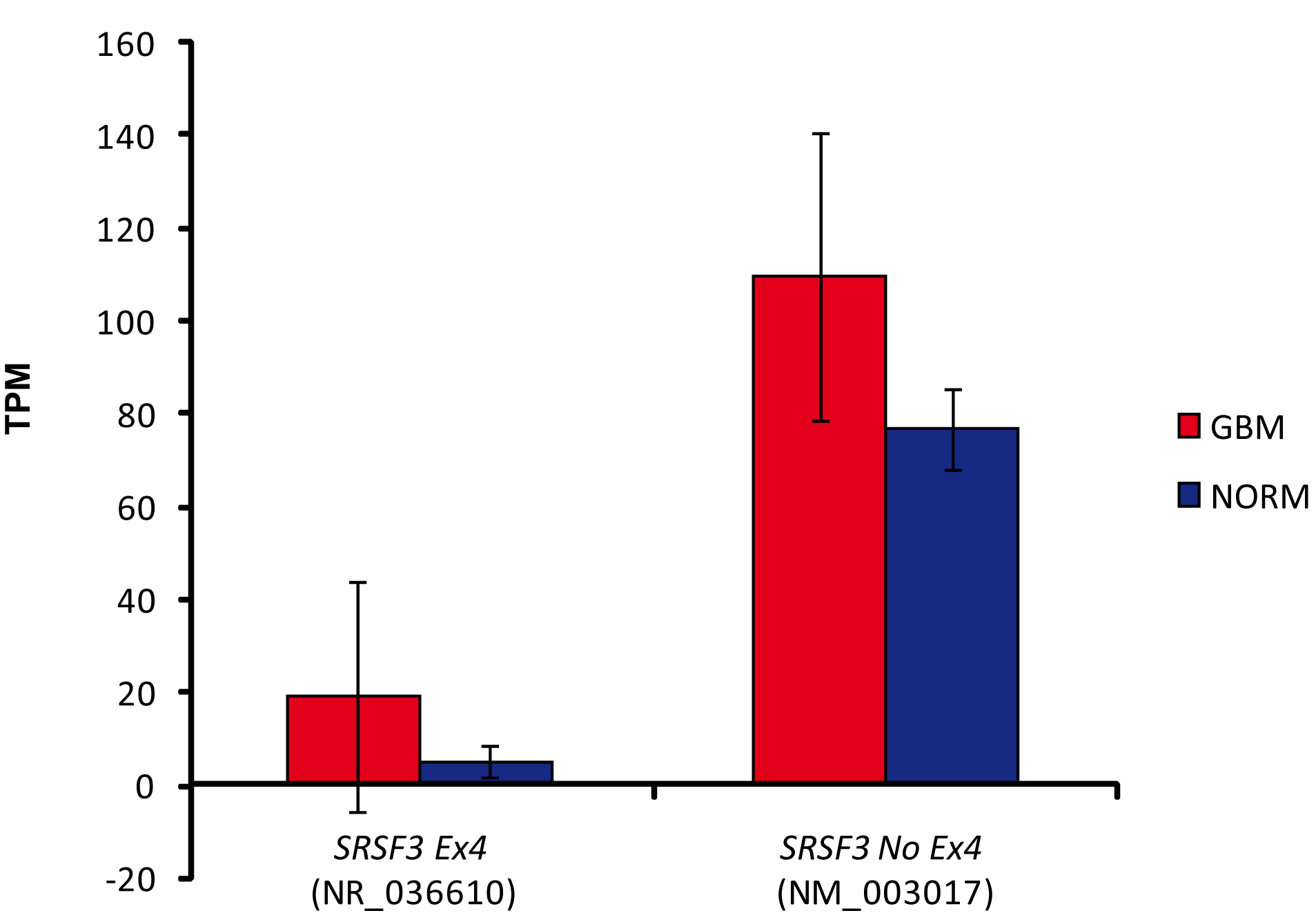
**

**Figure S6.** Expression of (mRNA) *SRSF3* isoforms in GBM and control samples (data from TCGA). Expression values are reported as Transcripts per Million (TPM) and data are represented as mean ± SD (n_GBM_ = 156, n_NORM_ = 5).


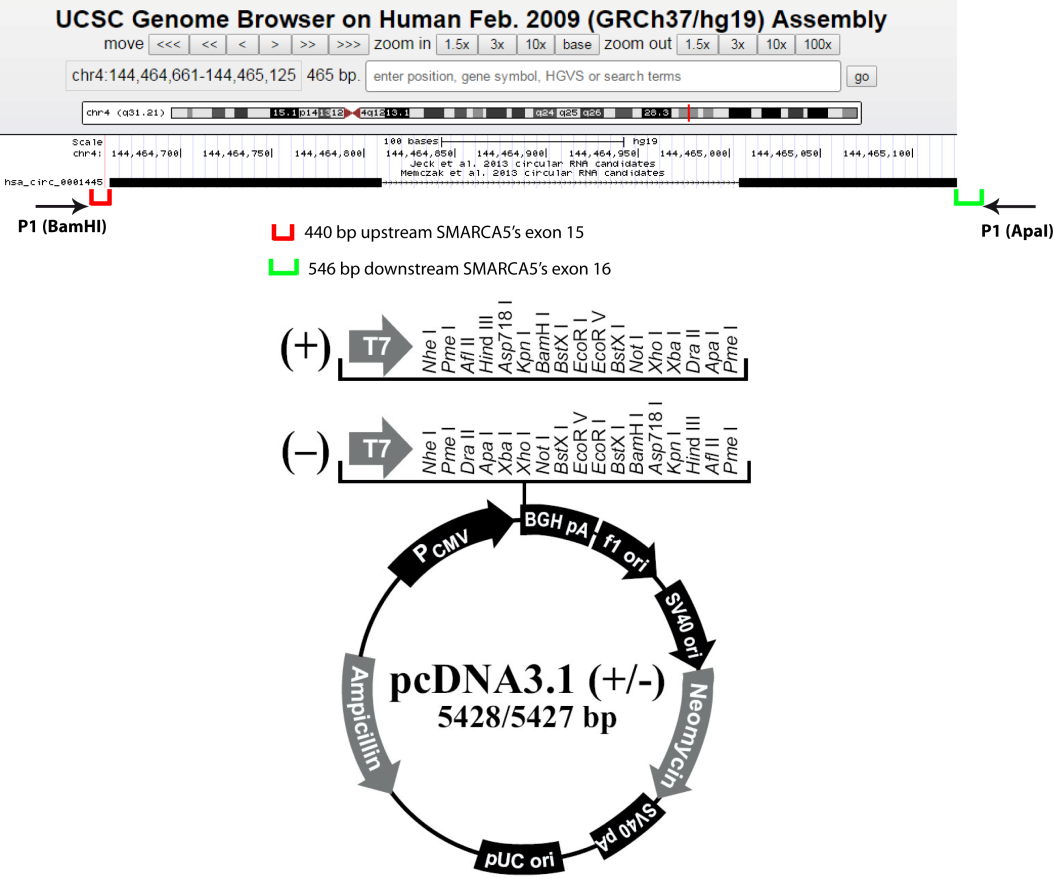


**Figure S7.** Schema of circSMARCA5’s cloning into the expression vector pcDNA3 (see Materials and Methods for details).
